# Terapia de Ressincronização Cardíaca em Cardio-Oncologia: Evidências do Benefício no Mundo Real em Doentes com Câncer e Insuficiência Cardíaca

**DOI:** 10.36660/abc.20250888

**Published:** 2026-07-31

**Authors:** Catarina Gregório, Andreia Magalhães, Beatriz Silva, Inês Araújo, Marta Vilela, Pedro Marques, João de Sousa, Miguel Nobre Menezes, Fausto J. Pinto, Manuela Fiuza

**Affiliations:** 1 Hospital de Santa Maria Faculdade de Medicina Universidade de Lisboa Lisboa Portugal CCUL@RISE Department of Cardiology, Hospital de Santa Maria (ULSSM), CAML, CCUL@RISE, Faculdade de Medicina, Universidade de Lisboa, Lisboa – Portugal; 2 Hospital Lusíadas Lisboa Lisboa Portugal Serviço de Cardiologia, Hospital Lusíadas Lisboa, Lisboa – Portugal

**Keywords:** Cardio-Oncologia, Insuficiência Cardíaca, Terapia de Ressincronização Cardíaca

## Introdução

A insuficiência cardíaca (IC) é uma importante causa de morbilidade e mortalidade entre doentes com câncer.^1^ A incidência de IC nesta população de alto risco tem vindo a aumentar, em parte devido aos efeitos cardiotóxicos associados às terapias oncológicas contemporâneas.^2^ Apesar dos avanços substanciais no tratamento oncológico e do crescente número de sobreviventes de câncer, a IC permanece como um importante determinante de desfechos clínicos adversos.^3^

A terapia de ressincronização cardíaca (TRC) é um tratamento estabelecido que melhora os sintomas e reduz a mortalidade em doentes com IC crónica.^4^ Contudo, os doentes com antecedentes de câncer foram frequentemente excluídos ou sub-representados nos principais ensaios clínicos de TRC, criando uma lacuna significativa na evidência sobre a eficácia e a segurança da TRC nesta população.^5^ Preocupações relacionadas com as comorbilidades, o prognóstico oncológico e o risco associado ao procedimento podem contribuir adicionalmente para a subutilização da TRC na prática clínica.

Deste modo, a evidência proveniente do mundo real é essencial para apoiar a tomada de decisão clínica e otimizar estratégias terapêuticas em doentes com IC relacionada ao câncer ou coexistente com disfunção ventricular esquerda (VE). Este estudo teve como objetivo avaliar a resposta clínica e ecocardiográfica à TRC em pacientes com IC e câncer ativo ou história prévia de neoplasia maligna.

## Métodos

Este estudo retrospectivo, unicêntrico, incluiu doentes adultos com câncer ativo ou história prévia de neoplasia maligna submetidos a implante de TRC entre 2011 e 2025 no Hospital de Santa Maria em Lisboa, Portugal. Câncer ativo foi definido como terapêutica oncológica em curso ou diagnóstico de câncer nos 12 meses anteriores. Sobreviventes de câncer foram definidos como doentes com antecedentes de neoplasia maligna em remissão há pelo menos 12 meses.^1,2^ Todos os doentes tinham recebido previamente pelo menos uma modalidade de tratamento oncológico (p.ex., quimioterapia, radioterapia, imunoterapia ou hormonoterapia) antes do implante da TRC. Nenhum doente foi submetido ao implante de TRC antes do diagnóstico de câncer ou do início do tratamento oncológico.

O implante de TRC foi realizado de acordo com as recomendações mais recentes da European Society of Cardiology para o tratamento da IC.^4^ Todos os doentes cumpriam os critérios de elegibilidade padrão para TRC, incluindo fração de ejeção do ventrículo esquerdo (FEVE) ≤ 35%, duração do QRS ≥ 130 ms e IC sintomática correspondente à classe funcional II-IV da New York Heart Association (NYHA), apesar do tratamento médico otimizado.^4^ A escolha do dispositivo, TRC com função de pacing (TRC-P) ou TRC com desfibrilhador (TRC-D), baseou-se nas indicações estabelecidas pelas recomendações e foi independente do status oncológico, embora as características clínicas individuais e as doentes tenham sido consideradas.

Dados demográficos e clínicos foram obtidos dos registos clínicos. Parâmetros ecocardiográficos, incluindo FEVE, volume telediastólico do ventrículo esquerdo (VTDVE) e volume telesistólico do ventrículo esquerdo (VTSVE), foram recolhidos no momento basal (antes do implante da TRC) e na última avaliação de seguimento disponível.

Os desfechos clínicos incluíram alterações na classe funcional NYHA, nos níveis da porção N-terminal do pró-peptídeo natriurético tipo B (NT-proBNP), mortalidade por todas as causas e hospitalizações por IC durante o seguimento. A resposta à TRC foi classificada de acordo com a redução relativa do VTSVE da seguinte forma: super-respondedores (redução ≥ 30%), respondedores (redução de 15%-29%), não respondedores (redução ≤ 14%) e progressores (aumento do VTSVE).^6^ Embora o estudo MADIT-CRT^6^ tenha definido resposta por meio de uma combinação de critérios volumétricos e sintomáticos, a presente análise baseou-se exclusivamente na redução do VTSVE, reconhecendo a heterogeneidade das definições de resposta à TRC entre os estudos.

As variáveis contínuas são apresentadas como média ± desvio-padrão ou mediana (intervalo interquartil [IIQ]), conforme apropriado, enquanto as variáveis categóricas são apresentadas como n (%). As alterações entre o momento basal e o seguimento foram avaliadas por meio do teste *t* para amostras emparelhadas ou do teste dos postos sinalizados de Wilcoxon para variáveis contínuas, e dos testes de McNemar ou qui-quadrado para variáveis categóricas. A correção de Bonferroni foi aplicada para comparações múltiplas envolvendo os principais desfechos ecocardiográficos. Um valor de p bicaudal <0,05 foi considerado estatisticamente significativo.

O estudo foi conduzido em conformidade com os padrões éticos institucionais e com os princípios da Declaração de Helsinque.

## Resultados

### Características basais

Um total de 51 pacientes foi submetido a implantação de TRC (idade média de 72,3 ± 10,2 anos; 63% do sexo masculino). As características basais estão resumidas na Tabela 1. A coorte apresentava uma elevada prevalência de fatores de risco cardiovascular, destacando-se a hipertensão arterial (84%), a dislipidemia (63%) e a diabetes (51%). Doença renal crônica e fibrilação atrial estavam presentes em 53% e 41% dos doentes, respectivamente.

**Tabela 1:** – Características demográficas e clínicas basais da população do estudo

Característica	Overall (n = 51)
**Sexo, n (%)**	
Masculino	32 (63)
Feminino	19 (37)
**Idade, anos, média ± DP**	72 ± 10
**Condições coexistentes, n (%)**	
Hipertensão arterial	43 (84)
Diabetes	26 (51)
Dislipidemia	32 (63)
Doença renal crônica (TFGe < 60 ml/min/1.73 m^2^)	27 (53)
Tabagismo ativo	17 (33)
Fibrilação atrial	21 (41)
**Neoplasia primária, n (%)**	
Câncer de mama	15 (29)
Câncer de próstata	12 (24)
Linfoma não Hodgkin	7 (14)
Câncer colorretal	6 (12)
Câncer de pulmão	2 (4)
Outros	9 (18)
**Doença metastática, n (%)**	3 (6)
**Tratamento oncológico prévio, n (%)**	
Quimioterapia	22 (43)
Radioterapia	10 (20)
Quimioterapia e radioterapia combinadas	12 (24)
Hormonoterapia	6 (12)
Imunoterapia	1 (2)
**Etiologia da IC, n (%)**	
CI	16 (31)
CIQ	17 (33)
MCD	15 (29)
CV	3 (6)
**Classe funcional NYHA basal, n (%)**	
II	35 (69)
III	15 (29)
**NT-proBNP, pg/ml, mediana (IIQ)**	4.148 (1.603-8.365)
**BRE, n (%)**	**35 (69)**
**Tipo de dispositivo, n (%)**	
TRC-P	24 (47)
TRC-D	27 (53)

BRE: bloqueio completo de ramo esquerdo; CIQ: cardiomiopatia induzida pela quimioterapia; MCD: Miocardiopatia Dilatada; CI: Cardiopatia Isquêmica; DP: desvio-padrão; IC: insuficiência cardíaca; IIQ: intervalo interquartil; NT-proBNP: porção N-terminal do pró-peptídeo natriurético tipo B; NYHA: New York Heart Association; TFGe: taxa de filtração glomerular estimada; TRC-D: terapia de ressincronização cardíaca com desfibrilador; TRC-P: terapia de ressincronização cardíaca com função de pacing; CV: cardiopatia valvular.

As neoplasias primárias mais comuns foram câncer de mama (29%), câncer de próstata (24%) e o linfoma não Hodgkin (14%), enquanto 6% dos pacientes apresentavam doença metastática. Todos os doentes tinham recebido tratamento oncológico previamente, incluindo quimioterapia (n = 22, dos quais 18 receberam antraciclinas), radioterapia (n = 10) ou tratamento combinado com quimioterapia e radioterapia (n = 12).

A cardiomiopatia induzida pela quimioterapia (CIQ) foi responsável por aproximadamente um terço dos casos. Os restantes doentes apresentavam cardiopatia isquémica (31%), miocardiopatia dilatada não relacionada com a quimioterapia (29%) ou cardiopatia valvular (6%).

No momento basal, a maioria dos doentes encontrava-se em classe funcional II (61%) ou III (29%) da NYHA sob terapêutica médica otimizada de acordo com as recomendações, incluindo inibidores da enzima conversora da angiotensina, bloqueadores dos receptores da angiotensina ou inibidores do receptor da angiotensina-neprilisina (78%), betabloqueadores (47%), antagonistas dos receptores mineralocorticoides (28%) e inibidores do cotransportador sódio-glicose tipo 2 (10%). A duração média do QRS foi de 151,1 ± 26,8 ms (mediana de 152,0 ms; IIQ 130,5-168,5), e o bloqueio completo de ramo esquerdo (BRE) estava presente em 69% dos doentes.

### Características do procedimento

Um total de 25 pacientes (53%) recebeu TRC-D, incluindo seis implantes em prevenção secundária, enquanto 24 pacientes (47%) receberam TRC-P. As complicações relacionadas com o procedimento foram incomuns e ocorreram em apenas um doente (1,9%), que desenvolveu um pequeno pneumotórax.

Os doentes com CIQ não diferiam significativamente dos doentes com outras etiologias de IC em relação à idade, comorbilidades ou terapêutica modificadora de prognóstico da IC (todos os valores de p > 0,05).

### Desfechos clínicos

Durante um seguimento médio de 45,3 ± 29,6 meses (IIQ 18-68), observou-se melhora significativa do estado funcional, com evolução para classes funcionais NYHA mais baixas (basal: NYHA II, 35 pacientes [68,6%]; NYHA III, 15 doentes [29,4%]; seguimento: NYHA I, 15 doentes[29,4%]; NYHA II, 23 doentes [45,1%]; NYHA III, 13 doentes [25,5%]; p = 0,005).

Os níveis medianos de NT-proBNP diminuíram de 4.148 pg/ml (IIQ 1.603-8.365) para 1.810 pg/ml (IIQ 370-6.149), correspondendo a uma redução relativa de 56% (p = 0,02) (Tabela 2).

**Tabela 2:** – Alterações nos parâmetros clínicos e ecocardiográficos após a terapia de ressincronização cardíaca

Parâmetro	Basal	Seguimento	Valor de p
**Parâmetros ecocardiográficos**			
FEVE, % (média ± DP) (n = 43)	29,7 ± 5,4	37,4 ± 11,7	< 0,001
VTDVE, ml (média ± DP) (n = 31)	180,0 ± 60,9	142,4 ± 57,4	< 0,001
VTSVE, ml (média ± DP) (n = 31)	128,7 ± 52,3	92,5 ± 51,6	< 0,001
**Parâmetros clínicos e laboratoriais**			
Classe funcional NYHA, n (%)			0,005
I	0 (0)	15 (29)	
II	35 (69)	23 (45)	
III	15 (29)	13 (26)	
NT-proBNP, pg/ml, mediana (IIQ)	4.148 (1.603-8.365)	1.810 (370-6.149)	0,020

DP: desvio-padrão; FEVE: fração de ejeção do ventrículo esquerdo; IIQ: intervalo interquartil; NT-proBNP: porção N-terminal do pró-peptídeo natriurético tipo B; NYHA: New York Heart Association; VTDVE: volume diastólico final do ventrículo esquerdo; VTSVE: volume sistólico final do ventrículo esquerdo.

### Desfechos ecocardiográficos e resposta à terapia de ressincronização cardíaca

Foram também observadas melhorias significativas nos parâmetros ecocardiográficos após a implantação de TRC (Tabela 2). Entre os 43 doentes com avaliações emparelhadas disponíveis, a FEVE aumentou de 29,7 ± 5,4% para 37,4 ± 11,7% (t[43] = −4,46; p < 0,001).

Entre os 31 doentes com dados volumétricos emparelhados disponíveis, o VTDVE diminuiu de 180,0 ± 60,9 ml para 142,4 ± 57,4 ml (p < 0,001), enquanto o VTSVE diminuiu de 128,7 ± 52,3 ml para 92,5 ± 51,6 ml (p < 0,001). Todas as comparações ecocardiográficas permaneceram estatisticamente significativas após a correção de Bonferroni.

Com base na redução relativa do VTSVE entre os doentes com dados volumétricos disponíveis (n = 31), 18 doentes (58%) foram classificados como super-respondedores, cinco (16%) como respondedores, quatro (13%) como não respondedores e quatro (13%) como progressores.

### Eventos clínicos durante o seguimento

Durante o seguimento, quatro doentes apresentaram terapias apropriadas do dispositivo, e 15 foram hospitalizados por IC. Não foram observadas diferenças significativas na terapêutica médica otimizada durante o seguimento entre respondedores e não respondedores (todos os valores de p > 0,05).

A mortalidade por todas as causas foi de 43,1% (n = 22), correspondendo a uma taxa anual de mortalidade de aproximadamente 11,4%, sendo as causas cardiovasculares responsáveis pela maioria dos óbitos (59%).

### Cardiomiopatia induzida por antraciclinas

Entre os 17 doentes com cardiomiopatia induzida pelas antraciclinas (CIA), observou-se uma melhoria significativa da classe funcional NYHA durante o seguimento (basal: NYHA II, 11 doentes [64,7%]; NYHA III, cinco doentes [29,4%]; NYHA IV, um doente [5,9%]; seguimento: NYHA I, oito doentes [33%]; NYHA II, oito doentes [60%]; NYHA III, um doente [7%]; p < 0,001).

Melhorias ecocardiográficas significativas também foram documentadas entre os 12 doentes com medidas volumétricas emparelhadas. O VTDVE diminuiu de uma mediana de 153,0 ml (IIQ 129,0-178,0) para 107,0 ml (IIQ 97,0-139,0; p = 0,013), enquanto o VTSVE diminuiu de 97,3 ml (IIQ 94,1-123,0) para 53,7 ml (IIQ 52,3-73,8; p = 0,006).

Com base na redução relativa do VTSVE, sete doentes foram classificados como super-respondedores, dois como respondedores, dois como não respondedores e um como progressor. Os doentes classificados como respondedores ou super-respondedores (n = 9) apresentaram uma melhoria mais acentuada da FEVE, com aumento mediano de 18,5% (IIQ 12%-25%), em comparação com 7,5% (IIQ 0%-16%) observado nos não respondedores e progressores (n = 3).

Todos os super-respondedores apresentavam perturbação de condução no momento basal, designadamente BRE, enquanto esse padrão foi observado em apenas dois dos três pacientes classificados como não respondedores/progressores.

Quando comparados com os doentes com outras etiologias de IC, aqueles com CIA apresentaram probabilidade semelhante de alcançar resposta à TRC (n = 30; p = 0,672). No entanto, a melhoria do estado funcional foi significativamente maior no subgrupo com CIA, com maior redução mediana da classe funcional NYHA (ΔNYHA 2,0 vs. 1,0; p = 0,008).

## Discussão

Nesta coorte do mundo real de pacientes com câncer e IC, a TRC associou-se a melhorias significativas da função ventricular esquerda, *remodeling* reverso e estado funcional. Os níveis de NT-proBNP diminuíram substancialmente, e a maioria dos doentes apresentou melhoria na classe funcional da NYHA, destacando os potenciais benefícios da TRC nesta população historicamente sub-representada. Adicionalmente, quando os critérios baseados nas recomendações para implantação de TRC em doentes com IC com fração de ejeção reduzida (ICFER) foram aplicados, indivíduos com diagnóstico atual ou prévio de câncer demonstraram resposta favorável, corroborando a aplicabilidade das indicações convencionais de TRC nesse contexto.^4^

Embora estudos prévios sugiram que apenas aproximadamente 21% dos doentes oncológicos elegíveis recebem TRC, estes resultados sugerem que o diagnóstico prévio de neoplasia não deve ser considerado uma contraindicação à terapêutica com dispositivos.^7^ Aproximadamente um terço de nossa coorte apresentava CIQ, predominantemente relacionada à exposição a antraciclinas. Nesse subgrupo, a TRC associou-se a uma melhoria sintomática e ecocardiográfica significativa, em consonância com os resultados do estudo MADIT-CHIC.^8^ Além disso, todos os super-respondedores e respondedores apresentavam perturbação da condução no período basal, reforçando o conceito de que a dessincronia elétrica, mais do que a lesão miocárdica isoladamente, constitui um importante determinante da resposta à TRC.

A TRC pode promover a recuperação miocárdica em doentes com disfunção ventricular esquerda associada à cardiotoxicidade através da correção da dissincronia mecânica, da melhoria da eficiência contrátil ventricular e da promoção do remodeling reverso.^5^ Além disso, a TRC provavelmente atua de forma sinérgica com a terapêutica modificadora de prognóstico da IC, mitigando a disfunção ventricular persistente e favorecendo a recuperação funcional.^9^ Estas observações estão em consonância com o recente posicionamento da Heart Failure Society of America (HFSA) em cardio-oncologia, que enfatiza o papel das terapias baseadas em dispositivos em pacientes selecionados com CIQ.^2^

As complicações relacionadas ao procedimento foram raras e limitadas a eventos de menor gravidade, sustentando e a segurança do implante de TRC em pacientes com câncer.^8^

As causas cardiovasculares foram responsáveis pela maioria dos óbitos, superando a mortalidade atribuída à progressão do câncer. Este achado reforça a importância da otimização do tratamento da IC através da terapêutica farmacológica orientada por recomendações e de intervenções apropriadas baseadas em dispositivos nesta população.

### Limitações do estudo

Diversas limitações devem ser reconhecidas. O desenho retrospectivo e unicêntrico, o tamanho relativamente reduzido da amostra e o acompanhamento ecocardiográfico incompleto limitam a generalização dos nossos resultados. Ainda assim, este estudo fornece evidência valiosa do mundo real que apoia o uso da TRC em doentes com ICFER e diagnóstico atual ou prévio de câncer. As análises de subgrupos, particularmente as que envolveram CIA, devem ser consideradas exploratórias devido ao número limitado de doentes. Além disso, uma pequena proporção de doentes recebeu terapêutica oncológica adicional após o implante da TRC, refletindo a presença de neoplasia ativa durante o seguimento. Consequentemente, não se pode excluir a possível influência de tratamentos oncológicos subsequentes sobre o *remodeling* ventricular e os desfechos clínicos. Por fim, as estimativas de mortalidade anualizada devem ser interpretadas com cautela devido à variabilidade no tempo de seguimento.

## Conclusão

Em conclusão, estes resultados sugerem que o diagnóstico atual ou prévio de câncer não deve constituir, por si só, um fator limitante à implantação de dispositivos de ressincronização cardíaca em doentes com ICFER que cumpram os critérios estabelecidos pelas recomendações. Estes dados sublinham a necessidade de uma abordagem multidisciplinar integrada, envolvendo cardiologistas, incluindo especialistas em dispositivos cardíacos, em estreita colaboração com oncologistas, de forma a individualizar a seleção dos doentes. A TRC pode proporcionar melhoria sintomática significativa, *remodeling* reverso e melhoria da qualidade de vida em doentes com neoplasias potencialmente tratáveis e expectativa de vida razoável. Estudos prospectivos multicêntricos são necessários para confirmar essas observações e aperfeiçoar a seleção dos doentes candidatos a esta terapia.
