# Supplementary material for: Terapia de Ressincronização Cardíaca em Cardio-Oncologia: Evidências do Benefício no Mundo Real em Doentes com Câncer e Insuficiência Cardíaca
Source: Arq Bras Cardiol. 2026 Jul 31;123(7):e20250888. [Article in Portuguese] doi: 10.36660/abc.20250888 (PMC13614024; doi:10.36660/abc.20250888)
Supplement: Translation

# Translation: Cardiac Resynchronization Therapy in Cardio-Oncology: Real-World Evidence of Benefit in Cancer Patients with Heart Failure

**DOI:** 10.36660/abc.20250888i

**Authors:** Catarina Gregório, Andreia Magalhães, Beatriz Silva, Inês Araújo, Marta Vilela, Pedro Marques, João de Sousa, Miguel Nobre Menezes, Fausto J. Pinto, Manuela Fiuza

**Affiliations:** 1 Portugal Department of Cardiology, Hospital de Santa Maria (ULSSM), CAML, CCUL@RISE, Faculdade de Medicina, Universidade de Lisboa, Lisboa – Portugal; 2 Portugal Serviço de Cardiologia, Hospital Lusíadas Lisboa, Lisboa – Portugal

**Keywords:** Cardio-Oncology, Heart Failure, Cardiac Resynchronization Therapy

## Introduction

Heart failure (HF) is a major cause of morbidity and mortality among patients with cancer.^1^ The incidence of HF in this high-risk population is increasing, partly due to the cardiotoxic effects associated with contemporary cancer therapies.^2^ Despite substantial advances in oncological treatments and the growing number of cancer survivors, HF remains a major determinant of adverse clinical outcomes.^3^

Cardiac resynchronization therapy (CRT) is an established treatment that improves symptoms and reduces mortality in patients with chronic HF.^4^ However, patients with a history of cancer have been frequently excluded or underrepresented in pivotal CRT trials, resulting in a significant evidence gap regarding the efficacy and safety of CRT in this population.^5^ Concerns related to comorbidities, cancer prognosis, and procedural risk may further contribute to the underutilization of CRT in clinical practice.

Real-world evidence is therefore essential to support clinical decision-making and optimize treatment strategies for patients with cancer-related or coexisting HF and left ventricular (LV) dysfunction. This study aimed to evaluate the clinical and echocardiographic response to CRT in patients with HF and either active cancer or a prior history of malignancy.

## Methods

This retrospective, single-center study included adult patients with either active cancer or a prior history of malignancy who underwent CRT implantation between 2011 and 2025 at Hospital de Santa Maria, Lisbon, Portugal. Active cancer was defined as ongoing anticancer treatment or a cancer diagnosis within the preceding 12 months. Cancer survivors were defined as patients with a history of malignancy in remission for at least 12 months.^1,2^ All patients had received at least one form of cancer therapy (e.g., chemotherapy, radiotherapy, immunotherapy, or hormone therapy) before CRT implantation. No patient underwent CRT implantation before cancer diagnosis or the initiation of oncological treatment.

CRT implantation was performed in accordance with the most recent European Society of Cardiology guidelines for HF management.^4^ All patients met standard eligibility criteria for CRT, including a LV ejection fraction (LVEF) ≤ 35%, QRS duration ≥ 130 ms, and symptomatic HF corresponding to New York Heart Association (NYHA) functional class II-IV despite optimal medical therapy.^4^ Device selection, CRT pacemaker (CRT-P) or CRT defibrillator (CRT-D), was based on established guideline indications and was independent of cancer status, although individual clinical characteristics and comorbidities were taken into consideration.

Demographic and clinical data were obtained from medical records. Echocardiographic parameters, including LVEF, LV end-diastolic volume (LVEDV), and LV end-systolic volume (LVESV), were collected at baseline (before CRT implantation) and at the most recent follow-up evaluation.

Clinical outcomes included changes in NYHA functional class, N-terminal pro-B-type natriuretic peptide (NT-proBNP) levels, all-cause mortality, and HF-related hospitalizations during follow-up. CRT response was classified according to the relative reduction in LVESV as follows: super-responders (≥ 30% reduction), responders (15%-29% reduction), non-responders (≤ 14% reduction), and progressors (increase in LVESV).^6^ Although the MADIT-CRT trial^6^ defined response using a combination of volumetric and symptomatic criteria, the present analysis was based exclusively on LVESV reduction, recognizing the heterogeneity of CRT response definitions across studies.

Continuous variables are presented as mean ± SD or median (IQR) as appropriate, whereas categorical variables are reported as n (%). Changes between baseline and follow-up were assessed using paired *t* tests or Wilcoxon signed-rank tests for continuous variables and McNemar or chi-square tests for categorical variables. Bonferroni correction was applied for multiple comparisons involving the primary echocardiographic endpoints. A two-sided p-value <0.05 was considered significant.

The study was conducted in accordance with institutional ethical standards and the principles of the Declaration of Helsinki.

## Results

### Baseline characteristics

A total of 51 patients underwent CRT implantation (mean age 72.3 ± 10.2 years; 63% male). Baseline characteristics are summarized in Table 1. The cohort exhibited a high burden of cardiovascular risk factors, with hypertension (84%), dyslipidemia (63%), and diabetes mellitus (51%) being the most prevalent. Chronic kidney disease and atrial fibrillation were present in 53% and 41% of patients, respectively.

**Table 1:** – Baseline demographic and clinical characteristics of the study population

Characteristic	Overall (n = 51)
**Sex, n (%)**	
Male	32 (63)
Female	19 (37)
**Age, years, mean ± SD**	72 ± 10
**Coexisting conditions, n (%)**	
Hypertension	43 (84)
Diabetes mellitus	26 (51)
Dyslipidemia	32 (63)
Chronic kidney disease (eGFR < 60 mL/min/1.73 m2)	27 (53)
Current smoker	17 (33)
Atrial fibrillation	21 (41)
**Primary malignancy, n (%)**	
Breast cancer	15 (29)
Prostate cancer	12 (24)
Non-Hodgkin lymphoma	7 (14)
Colorectal cancer	6 (12)
Lung cancer	2 (4)
Other	9 (18)
**Metastatic disease, n (%)**	3 (6)
**Previous cancer therapy, n (%)**	
Chemotherapy	22 (43)
Radiotherapy	10 (20)
Combined chemotherapy and radiotherapy	12 (24)
Hormone therapy	6 (12)
Immunotherapy	1 (2)
**HF etiology, n (%)**	
ICM	16 (31)
CICM	17 (33)
DCM	15 (29)
VHD	3 (6)
**Baseline NYHA functional class, n (%)**	
II	35 (69)
III	15 (29)
**NT-proBNP, pg/mL, median (IQR)**	4,148 (1,603-8,365)
**LBBB, n (%)**	35 (69)
**Device type, n (%)**	
CRT-P	24 (47)
CRT-D	27 (53)

CICM: chemotherapy-induced cardiomyopathy; CRT-D: cardiac resynchronization therapy defibrillator; CRT-P: cardiac resynchronization therapy pacemaker; DCM: dilated cardiomyopathy; eGFR: estimated glomerular filtration rate; HF: heart failure; ICM: ischemic cardiomyopathy; LBBB: left bundle branch block; NT-proBNP: N-terminal pro-B-type natriuretic peptide; NYHA: New York Heart Association; VHD: valvular heart disease.

The most common primary malignancies were breast cancer (29%), prostate cancer (24%), and non-Hodgkin lymphoma (14%), while 6% of patients had metastatic disease. All patients had previously received oncological treatment, including chemotherapy (n = 22, of whom 18 received anthracyclines), radiotherapy (n = 10), or combined chemotherapy and radiotherapy (n = 12).

Chemotherapy-induced cardiomyopathy (CICM) accounted for approximately one-third of cases. The remaining patients had ischemic cardiomyopathy (31%), non-chemotherapy-related dilated cardiomyopathy (29%), or valvular heart disease (6%).

At baseline, most patients were in NYHA functional class II (61%) or III (29%) and were receiving guideline-directed medical therapy, including angiotensin-converting enzyme inhibitors, angiotensin receptor blockers, or angiotensin receptor-neprilysin inhibitors (78%), beta-blockers (47%), mineralocorticoid receptor antagonists (28%), and sodium-glucose cotransporter 2 inhibitors (10%). Mean QRS duration was 151.1 ± 26.8 ms (median 152.0 ms; IQR 130.5-168.5), and left bundle branch block (LBBB) was present in 69% of patients.

### Procedural characteristics

A total of 25 patients (53%) received CRT-D, including six implanted for secondary prevention, whereas 24 patients (47%) received CRT-P. Procedural complications were uncommon and occurred in only one patient (1.9%), who developed a small pneumothorax.

Patients with CICM did not differ significantly from those with other HF etiologies regarding age, comorbidities, or HF therapy (all p > 0.05).

### Clinical outcomes

During a mean follow-up of 45.3 ± 29.6 months (IQR 18-68), functional status improved significantly, with a shift toward lower NYHA classes (baseline: NYHA II, 35 patients [68.6%]; NYHA III, 15 patients [29.4%]; follow-up: NYHA I, 15 patients [29.4%]; NYHA II, 23 patients [45.1%]; NYHA III, 13 patients [25.5%]; p = 0.005).

Median NT-proBNP levels decreased from 4,148 pg/mL (IQR 1,603-8,365) to 1,810 pg/mL (IQR 370-6,149), corresponding to a 56% relative reduction (p = 0.02) (Table 2).

**Table 2:** – Changes in clinical and echocardiographic parameters following cardiac resynchronization therapy

Parameter	Baseline	Follow-up	p-value
**Echocardiographic parameters**			
LVEF, % (mean ± SD) (n = 43)	29.7 ± 5.4	37.4 ± 11.7	< 0.001
LVEDV, mL (mean ± SD) (n = 31)	180.0 ± 60.9	142.4 ± 57.4	< 0.001
LVESV, mL (mean ± SD) (n = 31)	128.7 ± 52.3	92.5 ± 51.6	< 0.001
**Clinical characteristics and biomarkers**			
NYHA functional class, n (%)			0.005
I	0 (0)	15 (29)	
II	35 (69)	23 (45)	
III	15 (29)	13 (26)	
NT-proBNP, pg/mL, median (IQR)	4,148 (1,603-8,365)	1,810 (370-6,149)	0.020

LVEF: left ventricular ejection fraction; LVEDV: left ventricular end-diastolic volume; LVESV: left ventricular end-systolic volume; NT-proBNP: N-terminal pro-B-type natriuretic peptide; NYHA: New York Heart Association.

### Echocardiographic outcomes and response to cardiac resynchronization therapy

Significant improvements in echocardiographic parameters were also observed following CRT implantation (Table 2). Among the 43 patients with paired measurements, LVEF increased from 29.7 ± 5.4% to 37.4 ± 11.7% (t[43] = −4.46, p < 0.001).

Among the 31 patients with available paired volumetric data, LVEDV decreased from 180.0 ± 60.9 mL to 142.4 ± 57.4 mL (p < 0.001), while LVESV decreased from 128.7 ± 52.3 mL to 92.5 ± 51.6 mL (p < 0.001). All echocardiographic comparisons remained statistically significant after Bonferroni correction.

Based on the relative reduction in LVESV among patients with available volumetric data (n = 31), 18 patients (58%) were classified as super-responders, five (16%) as responders, four (13%) as non-responders, and four (13%) as progressors.

### Clinical events during follow-up

During follow-up, four patients experienced appropriate device therapy, and 15 required hospitalization for HF. No significant differences in guideline-directed medical therapy at follow-up were observed between responders and non-responders (all p > 0.05).

All-cause mortality was 43.1% (n = 22), corresponding to an annualized mortality rate of approximately 11.4%, with cardiovascular causes accounting for the majority of deaths (59%).

### Anthracycline-induced cardiomyopathy

Among the 17 patients with anthracycline-induced cardiomyopathy (AIC), a significant improvement in NYHA functional class was observed during follow-up (baseline: NYHA II, 11 patients [64.7%]; NYHA III, five patients [29.4%]; NYHA IV, one patient [5.9%]; follow-up: NYHA I, eight patients [33%]; NYHA II, eight patients [60%]; NYHA III, one patient [7%]; p < 0.001).

Significant echocardiographic improvements were also documented among the 12 patients with paired volumetric measurements. LVEDV decreased from a median of 153.0 mL (IQR 129.0-178.0) to 107.0 mL (IQR 97.0-139.0; p = 0.013), while LVESV decreased from 97.3 mL (IQR 94.1-123.0) to 53.7 mL (IQR 52.3-73.8; p = 0.006).

Based on the relative reduction in LVESV, seven patients were classified as super-responders, two as responders, two as non-responders, and one as a progressor. Patients classified as responders or super-responders (n = 9) demonstrated a greater improvement in LVEF, with a median increase of 18.5% (IQR 12%-25%), compared with 7.5% (IQR 0%-16%) among non-responders and progressors (n = 3).

All super-responders presented with baseline conduction abnormalities, specifically LBBB, whereas this pattern was observed in only two of the three non-responders/progressors.

When compared with patients with other HF etiologies, those with AIC showed a similar likelihood of achieving a CRT response (n = 30; p = 0.672). However, the improvement in functional status was significantly greater in the AIC subgroup, with a larger median reduction in NYHA class (ΔNYHA 2.0 vs. 1.0; p = 0.008).

## Discussion

In this real-world cohort of cancer patients and survivors with HF, CRT was associated with significant improvements in LV function, reverse remodeling, and functional status. NT-proBNP levels decreased substantially, and most patients experienced an improvement in NYHA functional class, highlighting the potential benefits of CRT in this historically underrepresented population. Notably, when guideline-based criteria for CRT implantation in HF with reduced ejection fraction (HFrEF) were applied, patients with a current or prior history of cancer demonstrated a favorable response, supporting the applicability of standard CRT indications in this setting.^4^

Although previous studies suggest that only approximately 21% of eligible patients with cancer receive CRT, our findings indicate that a history of malignancy should not be viewed as a contraindication to device therapy.^7^ Approximately one-third of our cohort had CICM, predominantly related to anthracycline exposure. In this subgroup, CRT was associated with significant symptomatic and echocardiographic improvement, consistent with findings from the MADIT-CHIC study.^8^ Furthermore, all super-responders and responders exhibited baseline conduction abnormalities, supporting the concept that electrical dyssynchrony, rather than myocardial injury alone, is a major determinant of CRT responsiveness.

CRT may facilitate myocardial recovery in patients with cardiotoxic injury by correcting mechanical dyssynchrony, improving ventricular contractile efficiency, and promoting reverse remodelling.^5^ In addition, CRT likely acts synergistically with contemporary guideline-directed HF therapies, mitigating ongoing ventricular dysfunction and supporting functional recovery.^9^ These observations are consistent with the recent Heart Failure Society of America (HFSA) cardio-oncology statement, which emphasizes the role of device-based therapies in selected patients with CICM.^2^

Procedural complications were rare and limited to minor events, supporting the feasibility and safety of CRT implantation in patients with cancer.^8^

Cardiovascular causes accounted for most deaths, exceeding mortality attributable to cancer progression. This finding underscores the importance of optimizing HF management through guideline-directed pharmacological therapy and appropriate device-based interventions in this population.

### Limitations of study

Several limitations should be acknowledged. The retrospective, single-center design, relatively small sample size, and incomplete echocardiographic follow-up limit the generalizability of our findings. Nevertheless, the study provides valuable real-world evidence supporting the use of CRT in patients with HFrEF and a current or prior history of cancer. Subgroup analyses, particularly those involving AIC, should be considered exploratory because of the limited number of patients. In addition, a small proportion of patients received further anticancer therapy after CRT implantation, reflecting ongoing active malignancy during follow-up. Consequently, the potential influence of subsequent oncological treatment on ventricular remodeling and clinical outcomes cannot be excluded. Finally, annualized mortality estimates should be interpreted with caution given the variability in follow-up duration.

## Conclusion

Overall, our findings support the use of CRT in patients with HFrEF regardless of a current or previous cancer diagnosis when standard guideline criteria are met. These results further highlight the importance of multidisciplinary collaboration between cardiologists, including device specialists, and oncologists in the management of this complex population. CRT may provide meaningful symptomatic improvement, reverse remodeling, and enhanced quality of life in patients with treatable malignancies and a reasonable life expectancy. Prospective multicenter studies are warranted to confirm these observations and further refine patient selection.
